# Young Carer Perception of Control: Results of a Phenomenology with a Mixed Sample of Young Carers Accessing Support and Unknown to Services

**DOI:** 10.3390/ijerph19106248

**Published:** 2022-05-20

**Authors:** Ed Janes

**Affiliations:** Wales Institute of Social and Economic Research and Data (WISERD), Cardiff University, Spark, Maindy Rd., Cathays, Cardiff CF24 4HQ, UK; janese3@cardiff.ac.uk

**Keywords:** young carers, phenomenology, hard-to-reach, identification, control, routine

## Abstract

Identification challenges have resulted in young carers research largely being conducted with those who access support. Positive and negative impacts have been evidenced but there remains little consideration of the wider population. This phenomenology defines young carers as a spectrum of children with different experiences and aims to study the larger group. Participants were recruited from schools and projects, resulting in a mixed sample of young carers who were accessing support but also those who were unknown to services. Participants attended three interviews that initially gathered data on their caring role and family circumstances, before focusing on their health and well-being in the context of change. All interviews were transcribed and analysed at a whole-text and in-depth level to identify shared understanding. A study of the wider spectrum enabled the emergence of perception of control over their caring responsibilities as key to routine development. Although high levels of control helped some participants manage their roles, threats to control were identified, including instability in the care receiver’s condition, excessive caring and medical tasks. The original findings demonstrate how researching the wider spectrum can aid understanding of problematic care, and highlights the importance of recruiting young carers as a hard-to-reach group.

## 1. Introduction

Thirty years of research has explored the lives of young carers, children who care for family members. Over that time the definition of young carers has varied significantly in individual pieces of research, particularly concerning their age, level of responsibility, and reason for care provision. This paper is part of the mixed methods Caring Lives study and defines the population in line with the recent paper by Joseph et al. [1] on future directions in research. Young carers are, therefore, a broad spectrum of children and young people under the age of 18 who provide some level of regular care to a family member due to an illness or disability that includes mental health and substance misuse issues.

This spectrum recognises the heterogeneity of young carers in terms of their experiences and impacts, with this having implications for past research, current understanding and how we research the population in the future. This study questions whether research to date is representative of the larger population that constitutes the spectrum, and returns to the challenge of how to identify young carers who are unknown to services.

This article presents findings from the Caring Lives study that aimed to identify and recruit young carers unknown to services from the school setting, and consider how their caring responsibilities impacted on their wider lives, mental health, and psychosocial wellbeing.

### 1.1. Trends in Young Carer Research

Early research by Loughborough University’s Young Carers Research Group [2,3] focused on the social issue of children and young people providing inappropriate responsibilities, sometimes without the support of other family members and services, and how these responsibilities affected their education and social opportunities. The research highlighted young carers as a group in need of support and they quickly gained a presence in policy and legislation [4] including the Carers (Recognition and Services) Act [5].

Over time research has become more specific with recent studies focusing on the impacts of caring for people with particular illnesses and disabilities, including Motor Neuron Disease [6] and AIDS [7], caring for siblings [8], and evaluations of respite camps [9] and interventions [10]. There is also an increasing focus on benefit-finding [11,12,13] as a response to the increasing concern raised by Olsen and Clarke (2003) [14] that early research was predisposed to identifying negative outcomes. In addition, after long-term challenges relating to the availability of quantitative data, the development of large-scale young carer survey projects and increasing inclusion of young carer indicators in cohort studies are enabling more reliable estimates of prevalence [15] and cross-sectional studies of young carers in comparison to non-caring peers [16,17,18].

There is also evidence of diversification with the 2017 and 2021 cross-national and comparative classifications of young carer awareness and policy response [19,20] indicating an increasing internationalization of research with studies in Africa, Europe, North America, Asia, and Australasia. The diversification also extends to age with a continuing focus on young adult carers [21], and the joint study of young carers and young adult carers together [22,23].

As a result of this expansion, literature reviews have highlighted an extensive range of negative impacts including anger, depression, and anxiety, as well as psychosocial benefits, such as independence, maturity, and confidence [24,25]. More recently, Janes et al. (2021) [26] conducted a realist synthesis of previous research to increase clarity concerning how positive and negative impacts vary depending on the individual circumstances of young carers and their families. The resulting model of young carers’ mental health and psychosocial wellbeing considered caring responsibilities mechanisms including inappropriate responsibilities, time spent caring, and level of care, and how triggering these mechanisms could improve outcomes for young carers. Other parts of the model focused on support mechanisms that moderate the impacts of caring responsibilities, and the development of young carer identities.

### 1.2. Young Carer Representation in Past Research

The young carer spectrum advocated by Joseph et al. [1] is part of a continuing move away from the traditional focus on ‘substantial care’. This was a key feature of early research [2,3] and coincided with challenges in identifying young carers, resulting in research largely being conducted through projects with children who were accessing support as a result of their roles. This led to criticism from the disability studies [27] and children’s rights sector [28] that the research only represented the experiences of one part of the young carer population and therefore produced weak evidence. Aldridge and Becker (1996) [29] argued that the methods used reflected the challenge of identifying young carers, but accepted the need to investigate the use of other methods.

This change can also be seen in current Welsh [30] and English [31] policy and legislation that do not define young carers as having responsibilities above a certain threshold, and partly explains the varying prevalence of young carer estimates. Early prevalence studies cited in Aldridge and Becker (1993) [2] and Becker et al. (1998) [32] utilized alternative methods due to the lack of available quantitative data. Estimates were typically under 1% but it was suggested that there could potentially be large numbers of ‘hidden’ young carers who were reluctant to be identified. More recent estimates using now available large scale quantitative data, deemed more reliable due to the larger sample size and the confidential nature of responses, appear to support this with prevalenceranging from 2–8% and as high as 12% [33]. However, amongst studies that did not specify ‘substantial care’ in research questions and, therefore, measured prevalence of the whole spectrum, the figures are higher again, including 5.1%, 5.8%, and 6.2% over a three year period for a single cohort [34] but also 12% [16,35], 13% [17], and 17% [36].

These increasing prevalence rates, as a result of both defining young carers as a spectrum and improved methods for estimation, have implications for qualitative research. Despite long-term recognition of the need to diversify methods to better identify young carers outside of support projects [29], there is little evidence of solutions being considered. Instead, qualitative research with known young carers in the project setting appears to have been normalized.

The result of this is that, as reflected in the model of young carers’ mental health and psychosocial wellbeing [26], knowledge of young carers largely reflects service users. These young carers access support, possibly due to negative impacts that resulted in the family seeking support or the child being identified while, in contrast, those not accessing projects may not do so because they have fewer responsibilities or because they are particularly keen to remain unknown to services. What is clear is that as prevalence estimates continue to rise, qualitative research with those accessing projects is increasingly unrepresentative of the wider young carer spectrum.

## 2. Materials and Methods

This study is the first qualitative research known to the author to seek to recruit young carers that were unknown to services (i.e., schools, health services, social services, and young carer projects), for participation in a longitudinal phenomenology. Schools were identified as the best approach to disseminate information to a large and highly representative group of children.

### 2.1. Recruitment

Recruitment of young carers unknown to services was expected to be challenging given the sensitivity of the topic and the need for confidentiality. Ethical approval was granted by Cardiff University’s School of Social Sciences Research Ethics Committee for a flexible three-stage approach that included working with each participating school to develop a confidential procedure in line with their policies and processes, engaging with pupils, and seeking the consent of young carers and their families.

Project recruitment began in August 2018 with 20 schools in South Wales approached to take part, and an additional 30 schools were contacted in early 2019. Individual meetings were held with 12 interested schools to discuss how the project could be facilitated confidentially in their setting, with four specific considerations paramount. First, a named point of contact was identified who would liaise with the families of interested pupils to arrange consent while maintaining confidentiality from the wider school. Second, schools identified the most suitable way to disseminate information to pupils with most opting for short assemblies that were delivered by the researcher and reinforced with age-appropriate information sheets. Third, the most suitable approach to contacting and seeking the consent of families was discussed. Lastly, the meetings discussed how confidentiality of the participants and the interview contents would be maintained. Eight schools progressed to sign up to the project.

Following information dissemination, seven pupils approached their school’s point of contact about participating, and were given an additional information sheet and consent form for a parent or family member. Consent was opt-in with explicit permission from a family member required for their child to be involved. This was received for five children who became participants. One child was excluded due to consent not being received. Consent was received for the final pupil in early 2020 but their involvement was not possible due to school closures as a result of the coronavirus pandemic.

Recruitment in the school environment was partially successful but recruitment was protracted and time-consuming, as considered in more detail in Janes (2022) [34]. Recruitment was extended in April 2019 to include young carer projects due to the time limitations of the project. The process of recruitment remained similar with the researcher meeting interested project workers, attending young carer sessions, and arranging for consent forms to be sent to the families of interested children.

#### 2.1.1. Participant Information

Table 1 presents a summary of the 10 participants. Five young carers recruited through their schools were not accessing any support at the time of their first interview and were largely unknown to services. In contrast, five were accessing young carer projects. Six participants were female and four male. Ages ranged from 11 to 16, and duration of care varied from 9 months to 11 years. Three participants were siblings. Nine were current carers with seven supporting their mothers and two supporting siblings. The final participant was a former young carer who had supported their mother prior to their death. All the care receivers had an illness or disability.

#### 2.1.2. Participant Retention

Interviews were held between March 2019 and August 2020, with six participants completing the three interviews. Although participants reported finding it useful to talk to an external person about their lives, two did not participate beyond the first meeting due to the sensitivity of the topic and change in their home circumstances. 

The majority of data had been gathered prior to the coronavirus pandemic, but the closure of services presented challenges as interviews had been organised through schools and young carer projects. Following receiving ethical approval to host the remaining four interviews online, two interviews were held remotely with Harry and Patrick, but two participants could not be contacted.

### 2.2. Phenomenological Interviews

The phenomenological approach was underpinned by the hermeneutical ideas of Gadamer (2004) [37]. He recognised that the ability to accurately understand a person’s perceptions of a phenomena is often limited by the researcher’s own experiences and beliefs, but argued that genuine conversations between the researcher and participants can enable the development of a shared understanding or ‘fusion of horizons’. Gadamer was particularly interested in how a person’s perception of a phenomena changes over time as a result of additional experiences, and a longitudinal approach was developed to study how participants’ views of the caring role evolved with new experiences and changing circumstances. This enabled comparison between participants but also consideration of change for individual participants over time [38].

Participants attended three interviews over a year-long period. Given the age of participants and the sensitivity of the topic, the interviews were designed to put participants at ease [39]. Interviews were held in participants’ school and project settings to ensure a familiar environment, and the use of creative methods and informal activities enabled the building of rapport over the multiple interviews. Interviews were limited to a maximum of 60 min and were semi-structured with participants encouraged to take control of the conversation. This enabled participants to direct the conversation towards their wider lives and several discussed other issues including bereavement, family substance misuse and feelings of abandonment.

The focus of the data collection varied across the three interviews. The development of a topic guide for the first interview was informed by the model of young carers’ mental health and psychosocial wellbeing [26]. The primary focus of the first interview was to gather contextual information relating to the young carer and their family, and questions related to caregiving responsibilities (the care receiver’s illness and the young carer’s responsibilities), support (assistance within the family, awareness by individuals and services of carer status, and support being accessed), and identity (perception of caring and choice). Creative methods included a modified ‘body in a box’ template of two bodies representing the young carer and the care receiver that they could write or draw on to answer questions, and rating scales for support sources. These creative activities were a precursor to more detailed discussion.

**Figure 1:**
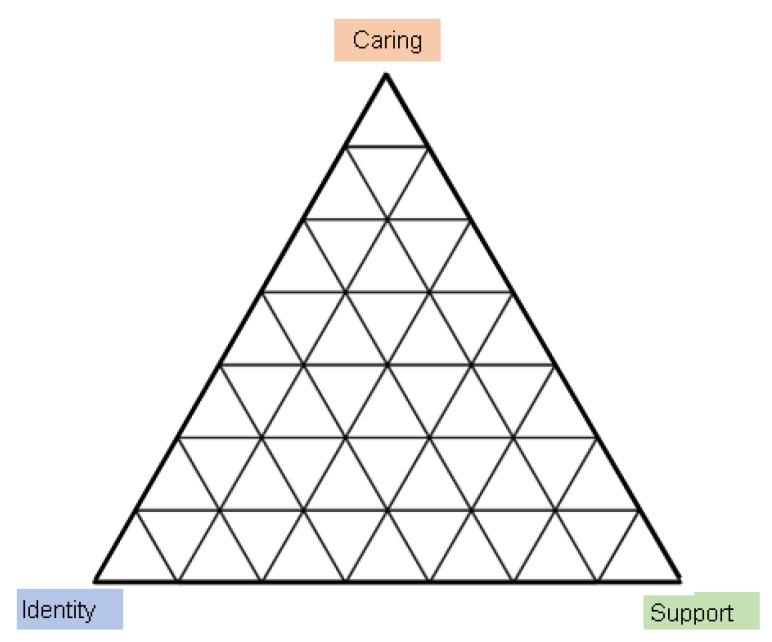
Participants were given an impact triangle to help them consider whether each impact was due to their caring responsibilities, support or their caring identity, or a combination of the three.

Transcriptions of each participant’s first interview enabled the researcher to prepare for the second meeting that began with a recap, additional questions to check clarity, and consideration of changing circumstances. These interviews were, therefore, more individualised, in order to help interpretation and understanding of the experiences previously discussed. The second interview then focused on their mental health and psychosocial wellbeing. Participants were initially asked to write down how they felt caring affected them, and then asked to choose from some pre-planned impact pairs (e.g., im-patient, un-stressed) that were based on the findings of previous research. All selected impacts were numbered. Participants were then given an impact triangle (Figure 1) that reflected the three domains of the young carers’ mental health and psychosocial wellbeing model. They placed each number in the triangle with the position reflecting whether the impact was due to their caregiving responsibilities, support, identity, or a combination of the three, before discussing why.

Transcription of the second interviews again informed the recap in the final meetings, as well as the developing of further questions to increase clarity further. The focus on change was more prominent in the final interviews, particularly for Harry and Patrick who had their final interview during the coronavirus pandemic, and participants actively considered their health and well-being in the context of changes in their wider circumstances.

#### Analysis

Following completion of data collection all first interview transcripts were analysed in the order that the meetings were held. Initial analysis was at a whole-text level to identify key content. This was followed by in-depth analysis using Nvivo 11 software to identify passages that demonstrated a shared understanding between the researcher and participants. The second, and then third, set of interviews were also analysed chronologically at a whole-text and in-depth level. Analysis of each participant’s later transcripts were, therefore, informed by their prior interviews, enabling an additional focus on how change in the participants’ health and wellbeing related to contextual change in their lives.

## 3. Results

This article concerns how perception of control affected management of caring responsibilities and aligns with the caring responsibilities domain of the young carers’ mental health and psychosocial wellbeing [26].

### 3.1. Perception of Control in the Lives of Young Carers

Perception of control across the ten participants varied and stability was often key to the development of routines that enabled positive management of the caregiver role alongside education and social opportunities. Several participants had responsibilities that were relatively stable due to a lack of change in the care receiver’s condition, including Angela, whose mother had increasingly learnt how to manage her MS (Multiple Sclerosis) in order to reduce tiredness and maximise her independence. She had a job that allowed her to work from home and would sometimes use a scooter when she went out, resulting in her *‘not getting better but learning to handle it better so it makes everything easier’* (I2).

As a result, Angela’s responsibilities had been relatively consistent during her two years as a young carer, and typically involved brief and regular tasks including domestic responsibilities and companionship. Angela recognised that her responsibilities were relatively minor compared to some young carers. She had been able to develop a routine where she prioritised her education, and could usually also plan her personal time and social life around these extra responsibilities:

*It’s that simple, if I’ve got to write an essay, I write an essay and I do my other stuff around it, I have my priorities and school comes first and then the other two *[caring and social]* kind of balance off each other… it makes it easier, because it’s like, I know if I’m going to have to do anything that evening, and if I’m not going to, it makes it easier to plan stuff.*

Angela (I3)

Harry had been a young carer for 11 years for his brother Sean who required constant care due to his autism. This care was provided within the family, and Harry’s responsibilities had evolved with his increasing age and Sean’s changing needs, from helping his brother settle at night to having a much more substantial role within the family routine. At the time of his first interview, Harry was responsible for *‘getting him up in the mornings, I do that the most out of everyone, because it’s sometimes hard for my parents when they’re also trying to help my other brother’*, preparing him for school, and caring after school while his parents were still at work. His parents were then the main carers at the weekend, giving Harry time for homework and to see his friends. 

Despite the increasing responsibilities over the 11 years, his responsibilities had stabilised in recent years and he described his responsibilities as *‘pretty much the same’* in his second interview. His final interview was held during the coronavirus pandemic, and impacts on family routine included Harry and Sean’s being home more as a result of their schools being closed, and their father being furloughed from work. Harry’s exams had also been cancelled, resulting in a changing life balance but, despite this, his caring responsibilities remained stable:

[During the pandemic] *I still had to do like some of the stuff, like get him out of bed in the morning and stuff, and like help him down to the car, if I need to go out shopping or stuff. There’s *[also]* been… help feeding and stuff, like feeding him, because usually he’s at school so but yeah apart from that, those things have been the same.*

Harry (I3)

Although Harry had a routine that was largely dependent on Sean’s needs and family member commitments, this stability largely worked for him. His substantial responsibilities could result in tiredness with Harry sometimes going *‘to bed late, wake up feeling really tired’* (I1) but, with the support of his family and local young carers project, he gained satisfaction from supporting Sean and reported an array of benefits including happiness, confidence, maturity, and independence:

*It makes me feel happy, because helping my brother makes me feel happy. And [not] lonely, I feel like I’ve got lots of people, lots of support, you know, helping me and stuff like that. Confident, I feel confident in my ability to care for my brother, and to not let him down I guess… And then helping me to feel mature, because it makes me feel independent.*

Harry (I1)

#### Positive Instability: Decreasing Responsibilities and Transition into the Carer Role

Although Angela’s control was the result of lower-level responsibilities and Harry’s due to a more substantial but stable role, Richard’s increasing control was the result of positive change in his family. He cared for his mother who was a recovering substance misuser and had mental health issues as a result of her addiction. Following her decision to give up drugs including *‘heroin when I was about five-ish and she went on substitutes… she’s given up the drink for two years and given up the weed for about a year and three months now’* (I1) he had been spending up to five hours a day supporting her through withdrawal symptoms. However, at the time of his first interview he was providing approximately two hours of predominantly domestic support each day. He recognised that his mother’s improved health reduced the need for care but he still chose to provide this lower level of support:

*I could easily just say ‘I give up. You go and do everything yourself’. I could easily go and say that, and my mother probably would panic and she probably would have a go at me. Things would get a little difficult for a bit but she would get herself around it all.*

Richard (I1)

Martin’s situation was significantly different to the other participants who were established young carers. In contrast, he had been a young carer for approximately nine months at the time of his first interview, due to the sudden onset of his mother’s aplastic anaemia which caused chronic fatigue. Martin had previously had chores but, as a new young carer, he had a fortnightly cycle of responsibilities that varied with his mother’s treatment and recovery weeks. His responsibilities varied over the fortnight but peaked at two or three hours each day of domestic responsibilities, physical support, and companionship:

*Normally we relax for like half an hour, and then I help her for an hour normally, half an hour, getting stuff before we start to cook tea, and then around half past four we start cooking tea and that’s about an hour, so about an hour and a half there, and then we eat tea, and then I normally help get her stuff ready, so that’s about another half an hour grabbing all her stuff and helping take it upstairs, and then normally I’m in her room for about half an hour, talking to her and just helping her around her room, closing her curtains,… I’d say about two and a half, three hours… It’s not much.*

Martin (I1)

**Figure 2:**
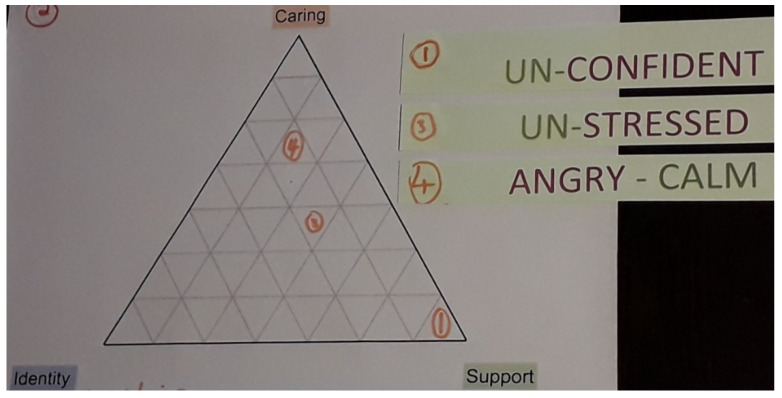
Martin’s frustration (impact 4) increased as a transitioning young carer.

Although Martin downplayed the amount of time that he spent caring, he was still becoming accustomed to his new role and, at the time of his first interview, Martin admitted increasing frustration (preferred to the term ‘angry’). In completing the impact triangle and explaining the placement of impacts, he attributed this frustration as largely due to his new caring responsibilities rather than the presence or absence of support or the development of a caring identity(Figure 2).

*It’s not good but I always like, I’m always annoyed. Like I value what I do but like I’m always annoyed and stuff, I have a really short temper. It’s like I used to but now, when my mum asks me to do stuff I’m always like ‘Argh’, and I know it’s not nice for my mum but I’m just, and when I wake up I’m a bit mean. But I, I don’t really know what I can do to stop that.*

Martin (I1)

The research followed Martin’s continuing transition, and by his third interview he reported increased confidence in his caring ability as a result of becoming accustomed to the amount of caring that he was providing. The increased confidence also had the effect of reducing his frustrations and he felt better able to balance caring and school, with the role becoming *‘routine, it’s just like not as stressful and like I’ve just gotten used to it’*. (I3)

### 3.2. Threats to Control of the Caring Role

The previous section focused on Angela, Harry, Richard, and Martin who were able to develop positive caring routines. They were largely either in control of their caring or experienced increasing control over the course of the year, and this resulted in a range of benefits, most notably for Harry despite his substantial role. This was not possible for the other six participants due to a number of threats to this control that included instability in the care receiver’s health, excessive responsibilities, night-time care, and the inclusion of medical responsibilities. These threats are considered next.

#### 3.2.1. Changing Care-Receiver Needs and Instability in the Caring Role

Central to Angela and Harry’s control of the caring role was stability but this was not possible when the care receiver’s health fluctuated over time. This was most noticeable for Thea, Lyra, and Lucy, three sisters who together cared for their mother and participated in the research as individuals. Their mother had been diagnosed with bipolar disorder following their father’s death. Initially sectioned for four months in 2015 and discharged, she was in hospital for a second time at the time of Lyra and Lucy’s first interview in August 2019, and had been released two months before Thea’s first interview in December 2019.

Thea detailed how their mother’s support needs were greatest when she was first released from hospital in 2015, and how *‘after two and a half, three years* [she had been able to] *to go out on her own, and she felt that was a big achievement. She was shopping all the time for herself’* (Thea, I1). Their mother’s needs increased after being sectioned the second time before decreasing again, and the sisters recognised that their responsibilities increased and decreased with her improving and deteriorating condition.

As the oldest sibling, Thea saw herself as her mother’s main carer and estimated that she was caring for six to seven hours a day at the time of her first interview:

*I help my mum when she gets ready in the mornings… so I’d say that’s about hour and a half getting her ready and getting myself ready. As soon as I come home, we usually do a daily shop because it’s not too much for my mum. So I’m there helping her with her shopping, and then I’m helping her then with the dishes. So about two hours then after school. Then we eat, so I help her with the food. It’s a good six/seven hours a day I would say.*

Thea (I1)

Thea felt this was ‘*a lot lower than from when she first come down* [in October 2019], *because I was proper, I was doing everything. Make sure nothing went wrong’* (I1), and her responsibilities had dropped further by her second interview in February 2020 when their mother was *‘productive, she likes to be independent, she likes to clean the house, she likes to make sure we’re getting ready for school, she likes to go out with some friends… it’s still a bit overwhelming for her, but she’s just getting back into the routine’* (I2).

**Figure 3:**
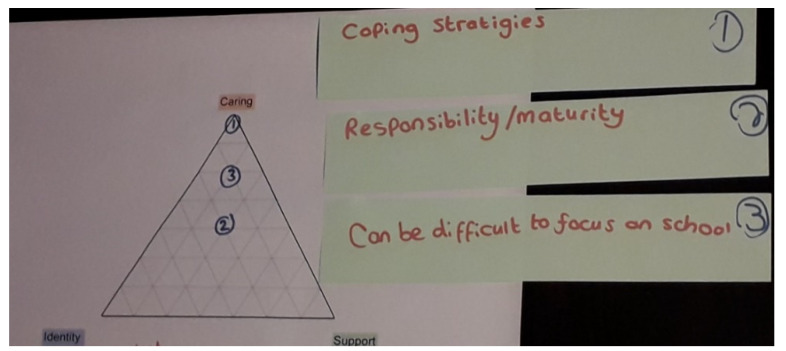
Thea struggled to balance caring with school and found that her extra responsibilities affected her school focus (Impact 3).

However, while these overall improvements in their mother’s health led to decreasing long-term responsibilities, the nature of her illness meant that her needs and their responsibilities fluctuated on a daily and weekly basis. Lyra described how *‘When my mum is depressed I find it kind of hard to make her do something, so like she doesn’t want to get up… then when she’s a maniac she’s kind of hard to calm down… but sometimes she will be in the middle where like she’s easy’* (I1). There were periods when their mother’s health was particularly poor and Lyra and Thea de-prioritised other parts of their lives to focus on caring. Lyra would attend school but *‘if I have homework or something I balance that when my mums asleep’* (I1), while Thea took sizeable periods off school to provide care, including at the time of her first interview. She was back in school at the time of her second meeting and increasingly focused on her education, but admitted that her caring responsibilities affected her concentration (Figure 3):

*When I come back into school, I got my head down like, focused as much as I could, because I didn’t want to let myself down, didn’t want to let myself fade away, it’s my last year, I’ve literally got about nine months left, there’s no point me messing it up now… It was hard at first, but I got back into the routine of, I’m doing well in school.*

Thea (I2)

Thea and Lyra’s social opportunities echoed their mother’s. When she was struggling and less active they would stay home with her and, when better, Thea would *‘choose to go out then’* (I1), while Lyra would *‘balance it because* [mother] *goes out with her friends and if I want to go out with my friends’* (I1).

As the oldest sibling Thea had also supported her sisters when her mother was in hospital. Between supporting her mother and sisters, the long-term and substantial responsibilities resulted in mental health issues, including anger, suicidal tendences, and self-harm, and Thea was receiving support from her local young carers project and social services. However, she also recognised that the effects were not from caring alone with Thea also affected by the death of her father and her feelings of abandonment following her mother being sectioned:

*Anger, definitely comes in, but not just my young carer role… I have tried to calm down a lot more, yeah so my anger isn’t as bad as it used to be, I don’t break things as much no more… I am proud of myself for being where I am now, because I was at a very low point when I finished school last year, I was extremely suicidal, tried to commit suicide three times last year, because of everything that happened.*

Thea (I2)

#### 3.2.2. Excessive and Night-Time Caring

Having considered instability in the care receiver’s condition as the first threat to perception of control, the second is excessive responsibilities. Although Harry was able to develop a positive routine to manage his substantial responsibilities, this was not always possible for participants with higher-level roles. Kirsty was a former young carer for her mother who had spina bifida and she had spent large amounts of time undertaking domestic tasks and giving companionship. Her caring did not interfere with school but the same was not true of her social opportunities as she increasingly prioritised caring for her mother over maintaining friendships. She explained how *‘for absolutely no reason I fell out with a lot of people… if something happened like, amongst my group of friends in school I couldn’t really care, couldn’t really care less but whereas something happened at home… lockdown panic mode’* (I2).

For some, these excessive responsibilities included night-time care that threatened control and routine development further. Patrick had been a young carer for his sister Sara since before the age of five and, similarly to Harry and his brother Sean, Sara needed constant support due to her cerebral palsy and learning difficulties. Patrick and his parents were extremely private to the extent of not seeking the support of wider family, and they would *‘rely on ourselves to keep us going because they* [wider family] *have their own lives that they should worry about rather than worrying about us’*. He had regular responsibilities within the family that included him helping Sara get ready for college in the morning and caring for four to five hours after school. He would then have more free time at the weekend when her parents were not at work. However, the additional responsibilities of monitoring Sara during the night resulted in Patrick waiting *‘until she is asleep and then I would just wait a couple of hours in case she does get up… she usually goes to bed at like ten… I’ll be up until like two’* (I2).

This combination of late nights and early mornings were causing long-term tiredness, but Patrick was fully focused on putting Sara’s needs first and seemed to have little consideration of his own health and well-being. His focus on caring led to the de-prioritisation of his homework, and he viewed his social life as happening within the school day:

*Social time’s done in school. Sister’s like after school and homework is trying to fit in anywhere possible like I’ll try and do homework before helping her but you never know when she needs help so it’s sort of keeping an eye on her whilst trying to do my work… sometimes it’s like I’ll forget about doing the homework because something else will crop up like she needs something and so it’ll be like the night before that I’m trying to rush trying to do it, so it is quite difficult.*

Patrick (I1)

Similarly to Harry, Patrick’s final interview was held during the coronavirus pandemic. Changes in his family included Sara shielding and Patrick not being in school, with his mother also furloughed and spending more time caring. This resulted in Patrick having fewer responsibilities and being *‘less tired now, because I’ve been doing half the work because there’s someone else doing it. Which is quite beneficial’* (I3). This, in turn, led to him having more time for *‘playing with my friends online, that’s been the best bit… I’ve been able to catch up with them more than I have ever before’* (I3). However, while his responsibilities eased, Sara became increasingly isolated as a result of the shielding, and Janes (2022) [34] considers how this led to a deterioration in their relationship.

Sophie was also a long-term young carer, having cared for her mother who had diabetes and a hearing impairment for nine years. Although her mother’s condition had remained largely unchanged, Sophie’s responsibilities had increased, from helping her mother communicate when she was aged four, to increasingly supporting her with her diabetes. She would monitor her mother every night due to a condition that caused her blood sugars to *‘just shoot straight up and she’ll wake up with really high blood sugars. But sometimes in the night she goes down really low… she doesn’t realise sometimes’* (I1). As a result, Sophie would be up until after midnight every day but this could be as late as four o’clock if her levels were abnormal. The amount of caring, and particularly the night-time responsibilities, resulted in tiredness and stress, and Sophie struggled to maintain the balance between caring and education. She shared her experiences of having to prioritise between caring and homework:

*I’ll have lots of homework to do and then my mum will need my help. And it’s like deciding which one’s more important… *[If undertake caring task]* I feel like I’ve helped my mum but then I’ve got to catch up on my own work… *[If do homework]* It makes me feel like I’ve done all my homework so I’m gonna be up to date, but then like, my mum might still be struggling, and like sometimes she’s like shaking as well so she can’t do it, and that’s why she asks me to do it… I feel like quite bad in the sense that I didn’t help her at the start.*

Sophie (I3)

#### 3.2.3. Medical Responsibilities

**Figure 4:**
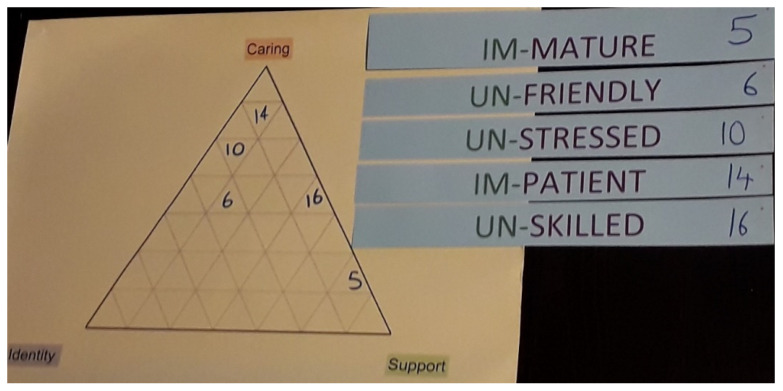
Unsuccessful medical care including attempts to stabilize blood sugar levels resulted in impatience (Impact 14) and a loss of control.

The final threat to control was the provision of medical care, and this also concerns Sophie as the only participant with regular medical responsibilities. In addition to monitoring her mother, Sophie would occasionally administer injections or more often force feed her mother when her blood sugar levels had spiked or collapsed. Sophie had been carrying out these responsibilities for several years without professional training, and the development of medical skills through experience had helped her to remain unstressed most of the time. However, maintaining this control was difficult when her attempts to manage her mother’s blood sugar levels were unsuccessful, and she would become distressed, frustrated, and impatient (Figure 4).

*When you test* [her blood] *it will start to show an improvement and she’ll start to feel better. If it doesn’t then you can get really, really impatient and angry about it… she had a hypo, I think it was last month or something, and it went down to like two or something and my mum didn’t wanna like, she didn’t wanna eat, so I was like force feeding her to eat, and then she was like, she just didn’t want to do anything, and then she was getting really hot and sweaty, and she had two or three cups of orange juice and two Twirls and it still took her a good 20 min for it to get back to a reasonable level that she could then go back to sleep.*

Sophie (I2)

Sophie was recruited from her school and talked about privacy and how her ‘*mum doesn’t want me getting like, a lot of attention for doing these things. Like, not that it’s bad or anything, but like if, like if I come in* [to school] *late and people just ask why I come in late’* (I2). Despite this, at the time of her first interview she had been identified by her mother’s diabetic nurse. This led to significant change for Sophie and her family as, over the course of the year, her mother was fitted with new equipment that reduced her needs and Sophie’s caring responsibilities. Janes (2022) [34] considers the support that was provided in more detail, and the positive impacts of the intervention on Sophie and her family.

## 4. Discussion

The introduction to this article revisited the challenge of identification in young carers research and argued that current understanding largely reflects those who access support, likely as a result of high-level responsibilities. This informed the innovative approach used in this study that sought to identify young carers in the school setting for involvement in a phenomenology.

Although recruitment challenges eventually resulted in this being expanded to include young carer projects, the sample still represented a wider range of experiences than in previous research. All five participants recruited from young carer projects had substantial responsibilities. In contrast, the five recruited from schools had responsibilities ranging from low to very high and, with the exception of Sophie who had recently been identified by a medical professional, they were unknown to services. There was also evidence of greater privacy in these five families.

Involvement of this wider range of young carers enabled the emergence of perception of control as a key factor in whether young carers are able to manage their responsibilities. This control was sometimes the result of stable, lower-level responsibilities caring for family members that were often largely able to manage their illnesses, but other young carers with more substantial but still stable responsibilities had also developed routines that worked for them. There was also evidence of increasing control over time for young carers with decreasing responsibilities, or for recently transitioned young carer as they became accustomed to their role.

The research also focused on the challenges that participants were facing. These were often similar to those identified in previous research but were reframed as threats to the control that other young carers had. First, whereas high levels of control were linked to care receiver stability, reduced control was often driven by ongoing change in the care receiver’s condition and needs [40,41]. This was most notable for Thea and Lyra, and their inability to develop a routine for managing their responsibilities alongside their education and social lives was clearly problematic as their mother’s illness improved and deteriorated over time.

A second threat concerned the amount of time spent caring [42] as several participants with greater responsibilities had problematic routines. In particular, the study highlighted the challenges of night-time caring and early morning responsibilities that combined to interfere with education and social opportunities and cause detrimental impacts. Finally, Sophie’s experiences of monitoring her diabetic mother’s blood sugar and attempting to stabilise her spiking levels reinforced the findings of Kavanaugh (2014) [43] on the inappropriateness of medical responsibilities. However, the contrast between Sophie struggling with these urgent caring tasks but managing her wider responsibilities evidenced how perception of control varies not only between young carers but also for individuals over time.

### Limitations and Opportunities for Further Work

Although the longitudinal focus of the study enabled an in-depth focus on how the impacts of caring varied over time, the sample was limited to 10 young carers. As a result, further study focusing overtly on control in a larger group of young carers is needed. In particular, past research has highlighted the inappropriate roles of young carers with intimate caring responsibilities, such as toileting or bathing [3,40], but this was not possible to study as none of the participants regularly provided these tasks. In addition, the study did not set out to investigate young carers during the coronavirus pandemic. While a detailed understanding of two young carers whose responsibilities were little affected or reduced during the pandemic is demonstrated, further research is needed into the experiences of other young carers during the pandemic. 

Participants also included a transitioning young carer who developed increasing control as they become more accustomed to their responsibilities. There is little evidence of research on children as they transition into a carer role, possibly because those who participate in research through projects are already established young carers, but there is considerable potential to study perception of control amongst newer young carers.

Although there is clear potential for further research on the wider young carer spectrum, this requires the improved identification of young carers that are unknown to services. An original aspect of this qualitative study was the recruitment of young carers in the school setting, and while this was partially successful the process was intensive and protracted. There is therefore a significant need for a methodological focus on how to work with young carers as a hard-to-reach group. With young carers increasingly being defined as all children with caring responsibility for a family member due to illness or disability this should also be of interest to the wider research field, otherwise research conducted through projects will only become less representative of this wider young carer spectrum.

## 5. Conclusions

Previous research has evidenced a range of positive and negative impacts of caring by children, and how the overall effects can be detrimental, marginal, or positive. This study investigated the experiences and impacts of a broader range of young carers than in most research, resulting in original findings on how perception of control can affect young carers’ ability to manage their caring responsibilities. Perception of control offers an alternative way to understand the wider young carer spectrum and also suggests that studying the comparatively manageable responsibilities of the larger group can aid our understanding of more problematic caring. This raises questions over the needs of all young carers, and whether the support provided by different sources (family, friends, community, projects, and mainstream services) should potential vary for different parts of the young carer spectrum.

## Figures and Tables

**Table 1:** Participant sample characteristics.

				Demographics		Care Receiver Details		Caring Details (at Interview 1)		
No	Setting	Interviews Completed	Name	Sex	Age	Care Receiver	Reason for Need	Duration as Young Carer	Level of Care	Key Responsibilities
1	School	3	Sophie	F	13	Mother	Diabetes, hearing impairment	9 years	Main	Medical care (force feeding, injections to stabilize blood sugar levels); companionship; translation
2	School	3	Angela	F	13	Mother	MS	2 years	Main	Companionship; domestic responsibilities
3	School	3	Kirsty	F	14	Mother *	Spina bifida, hydrocephalus, epilepsy	5 years	Joint	Domestic responsibilities; companionship
4	School	3	Martin	M	13	Mother	Aplastic anaemia	9 months	Main	Physical support; domestic responsibilities
5	Project	1	Lyra **	F	13	Mother	Bipolar disorder	4 years	Joint	Domestic responsibilities
6	Project	1	Lucy **	F	11	Mother	Bipolar disorder	2 years	Joint	Domestic responsibilities; nursing
7	Project	3	Harry ***	M	16	Brother	Autism	11 years	Joint	Getting up in the morning; physical support; taking to school; caring after school
8	School	3	Patrick ***	M	14	Sister	Cerebral palsy; learning difficulties	9 years	Joint	Physical support; monitoring
						Grandfather *	Old age, dizziness, confusion			Domestic responsibilities
9	Project	2	Richard	M	16	Mother	Former substance misuse; mental health	11 years	Main	Emotional support; domestic responsibilities
10	Project	2	Thea **	F	16	Mother	Bipolar disorder	4 years	Main	Companionship; nursing; domestic responsibilities

* Care receiver deceased at time of first interview. ** Participants were siblings. *** Third interview was online due to coronavirus restrictions.

## Data Availability

Due to the sensitive nature of the research and the challenges of recruiting young carers unknown to services, the research did not seek the consent of families to share data.
